# How do climate-linked sex ratios and dispersal limit range boundaries?

**DOI:** 10.1186/1472-6785-14-19

**Published:** 2014-07-10

**Authors:** Maria Boyle, Lisa E Schwanz, Jim Hone, Arthur Georges

**Affiliations:** 1 Institute for Applied Ecology, University of Canberra, Canberra ACT 2601, Australia; 2 School of Biological, Earth and Environmental Sciences, University of New South Wales, Sydney NSW 2052, Australia

**Keywords:** Dispersal, GSD, Population dynamics, Range limits, Reptiles, Sex ratio, TSD

## Abstract

**Background:**

Geographic ranges of ectotherms such as reptiles may be determined strongly by abiotic factors owing to causal links between ambient temperature, juvenile survival and individual sex (male or female). Unfortunately, we know little of how these factors interact with dispersal among populations across a species range. We used a simulation model to examine the effects of dispersal, temperature-dependent juvenile survival and sex determining mechanism (temperature-dependent sex determination (TSD) and genotypic sex determination (GSD)) and their interactions, on range limits in populations extending across a continuous range of air temperatures. In particular, we examined the relative importance of these parameters for population persistence to recommend targets for future empirical research.

**Results:**

Dispersal influenced the range limits of species with TSD to a greater extent than in GSD species. Whereas male dispersal led to expanded species ranges across warm (female-producing) climates, female dispersal led to expanded ranges across cool (male-producing) climates. Two-sex dispersal eliminated the influence of biased sex ratios on ranges.

**Conclusion:**

The results highlight the importance of the demographic parameter of sex ratio in determining population persistence and species range limits.

## Background

For many organisms, particularly ectothermic animals, individual traits and demographic parameters are linked to temperature, such that ranges could be largely determined by abiotic factors [1,2]. Abiotic factors are often incorporated into the most simple modelling approaches (for example, climate envelope modelling) used to predict the future distributions of organisms [3]. However, there is increasing evidence [3-8] that fast evolutionary changes, dispersal and population dynamics are among the factors that are equally as important as abiotic factors in determining species ranges [3,9]. For example, it has been demonstrated that population dynamics and dispersal can alter a species responses to shifting climates [3]. In particular there can be a considerable lag between climate becoming suitable in any one place and colonisation by a species through dispersal [3]. Across a species’ range, dispersal can play a key role by expanding ranges outside of a thermal limit (i.e. creating sinks,[10]), [Harts A, Schwanz L, Kokko H: Demography can favour female-advantageous alleles. P R SOC B. submitted], and preventing local adaptation [2]. These issues have gained new imperative for understanding range change in the context of climatic warming.

Colonisation and extinction rates may change spatially due to environmental gradients in habitat characteristics (for example, temperature, rainfall, or humidity) [11]. The role of temperature in demography and range limits may be especially important for species with temperature-dependent sex determination (TSD; [12,13]). In these species, individual sex (male or female) is permanently determined during embryonic development by incubation temperature [14]. The proportion of new hatchlings in a population that are male (the Cohort Sex Ratio, or CSR) often shows wide geographical variation associated with climatic or microclimatic variation and their effects on nest temperatures [15,16]. Numerous studies have predicted that climate warming will skew the cohort sex ratios of reptiles towards females (or males in the tuatara) with negative consequences for population persistence (e.g. [12,15,17-22]). Yet we know very little about how sex ratios in populations of reptiles with TSD limit range boundaries. There has been at least one attempt to explain the geographical distribution of TSD species based on sex ratios, suggesting that imbalanced sex ratios (and marginal habitats) limit population growth at range boundaries [13]. It was proposed that balanced sex ratios are located at range centres (or favourable habitats), and skewed sex ratios at more marginal habitats [13].

The importance of biased sex ratios in limiting the ranges of species with TSD will depend on a few interacting factors. First, climatically-linked juvenile survival may also play a role in determining range boundaries, as eggs only survive at certain temperatures [16,23] [Boyle M, Hone J, Schwanz L, Georges A: Under what conditions do climate-driven sex ratios enhance versus diminish population persistence? Ecology and Evolution. submitted]. Second, the extent to which female-biased sex ratios limit population persistence will depend on how ‘limiting’ males are (i.e. the dependence of female fecundity on male abundance, [Boyle M, Hone J, Schwanz L, Georges A: Under what conditions do climate-driven sex ratios enhance versus diminish population persistence? Ecology and Evolution. submitted] [24]. If male density is not strongly limiting to female fecundity because of polygyny, sperm storage and sex-specific breeding intervals [25-27], female-biased populations will have enhanced population growth and persistence [Boyle M, Hone J, Schwanz L, Georges A: Under what conditions do climate-driven sex ratios enhance versus diminish population persistence? Ecology and Evolution. submitted] [28].

Finally, dispersal among populations may bring the rare sex into biased populations, rescuing them from the risk of demographic collapse and potentially leading to expanded ranges. Dispersal by male hatchlings is thought to have an important role in facilitating population persistence in increasingly feminised populations [29]. However, most studies of the effects of climate warming on sex ratios are of isolated populations, and no consideration has been given to the role of dispersal, or the interaction between climatically-linked sex ratios and sex-specific dispersal in driving population dynamics. For example, in many TSD species dispersal may be inefficient for range expansion under climate change [16]. This suggests that the sensitivity of range limitation to dispersal distance should be examined.

Dispersal in reptiles is thought to be primarily male-biased, although data are sparse and alternative dispersal tendencies (female-biased dispersal or dispersal by both sexes) have not been well investigated [30,31]. For example, male-biased dispersal has been demonstrated in many species of lizards [32,33], snakes [31,34,35], marine turtles [36-39], and in some freshwater turtles [40-42]. Female dispersal has been demonstrated only in an alpine lizard (*Niveoscincus microlepidotus*) [30].

There are no documented studies of two-sex dispersal in reptiles. A theoretical analysis of two-sex dispersal tendency found it enhanced population persistence in marginal habitats as gene flow through dispersal occurred in both directions [43]. This analysis included benefits associated with choosing habitats to maximize environmental quality and a reduction in inbreeding. However, because there was no population variation in sex ratio, it did not explore the importance of dispersal in recruiting a rare sex into a population.

In this paper, we explore the role of dispersal in determining the extent of population persistence and range limits in species of reptiles with the common TSD pattern 1A (females produced at higher temperatures) and compare it to results for species with genotypic sex determination (GSD). We do this across populations that vary spatially, but not temporally, in air temperature. Importantly, we include in this analysis 1) the potential for male limitation on female fecundity when males are rare, and 2) the additional effect of temperature on juvenile survival.

## Methods

### Simulation model

A matrix (100 rows by 100 columns) of 10,000 populations was distributed across a continuous air temperature gradient, with each column assigned a temperature from 18°C to 33°C. Each population in the matrix was initiated with 100 adult males and 100 adult females (for both TSD and GSD species). We projected these populations in a simulation to determine range limits under different scenarios of dispersal and temperature-dependence of sex ratios. Within each population, simulated population operations approximated logistic growth, including density-dependent juvenile survival [Boyle M, Hone J, Schwanz L, Georges A: Under what conditions do climate-driven sex ratios enhance versus diminish population persistence? Ecology and Evolution. submitted].

In each time step, offspring were first produced. The total number of offspring produced was the product of the number of adult females in the population and per-female fecundity, which was subject to male limitation (see Male Limitation). The number of offspring in each population that were male was sampled using a random binomial distribution (and, hence sex ratio was stochastically determined), given the total number of offspring and the population-specific sex ratio probability of producing a male, *p*. This sex ratio probability depended on population air temperature for TSD scenarios, but was a constant 0.5 for GSD scenarios (see Cohort sex ratio). The number of offspring that recruited as adults into their natal population or a non-natal population (survived to and bred at age 1) depended on temperature-dependent juvenile survival (see Juvenile survival) followed by dispersal (see Dispersal function). The number of adults surviving to the next time step was sampled using a random binomial distribution given the initial number of adults and a constant probability of survival (*s* = 0.95 for both males and females; average life expectancy of 20 years). There was no adult dispersal.

The population cycle was iterated 1,000 times. Throughout the simulation, populations were counted as extinct if they had either zero adult males or zero adult females, and were excluded from reproduction in that round. Each simulation was replicated ten times and the averaged numbers of extant populations were plotted in ten temperature intervals, each of 1.5°C in width, in the range of temperatures in the population matrix. Simulations were performed with MATLAB R2012b.

### Male limitation (*B*)

In our simulation, per-female fecundity, *B*, was an integer that was sampled using a random binomial distribution based on the probability of fertilisation of a female, Pr{*fert}*, with a maximum value of *B*_*max*_:

(1) $$B=B_{\max}x\;\mathrm{Pr}\left\{\mathrm{fert}\right\}$$

Pr{fert} was described as a function of adult sex ratio (ASR), or the proportion of adults in the population that are male, [24]:

(2) $$\mathrm{Pr}\left\{\mathrm{fert}\right\}=\frac{\mathrm{ASR}}{\mathrm{ASR}+b}\;$$

**Figure 1:**
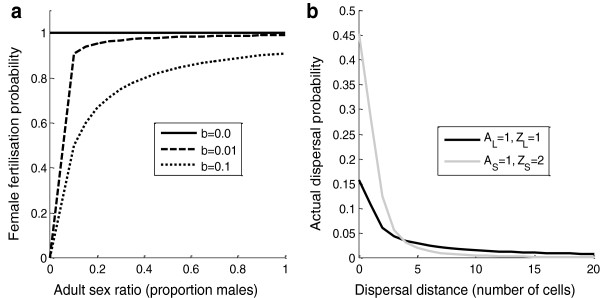
**Adult sex ratio and fat-tailed dispersal kernels. (a)** The female fertilisation probability as a function of adult sex ratio (ASR = M/(M + F)). The different lines represent different sensitivities of fertilisation probability to changes in the ASR (after Rankin and Kokko 2007). **(b)** Actual probability of an individual moving a certain number of cells (dispersal distance) across a population matrix, according to two fat-tailed dispersal kernels (equation 3). ‘Large’ dispersal has parameters A_L_ = 1 and Z_L_ = 1 (black line) and ‘Small’ dispersal has parameters A_S_ = 1 and Z_S_ = 2 (grey line), where L = Large, and S = Small.

The shape parameter for equation 2, *b,* represents the relative strength of male limitation on female fecundity. Persistence of isolated TSD populations in female-producing climates is highly sensitive to this parameter [Boyle M, Hone J, Schwanz L, Georges A: Under what conditions do climate-driven sex ratios enhance versus diminish population persistence? Ecology and Evolution. submitted]. However, there are few empirical data available to estimate a likely value. Given the high degree of polygamy and sperm storage in reptiles [25-27], it is unlikely that males are strongly limiting on female fecundity. For this reason, we chose an intermediate level of male limitation (*b* = 0.01) where males only become strongly limiting to female fecundity when they fall below approximately 10% of the adult population (Figure 1a).

### Cohort sex ratio (*p*)

**Figure 2:**
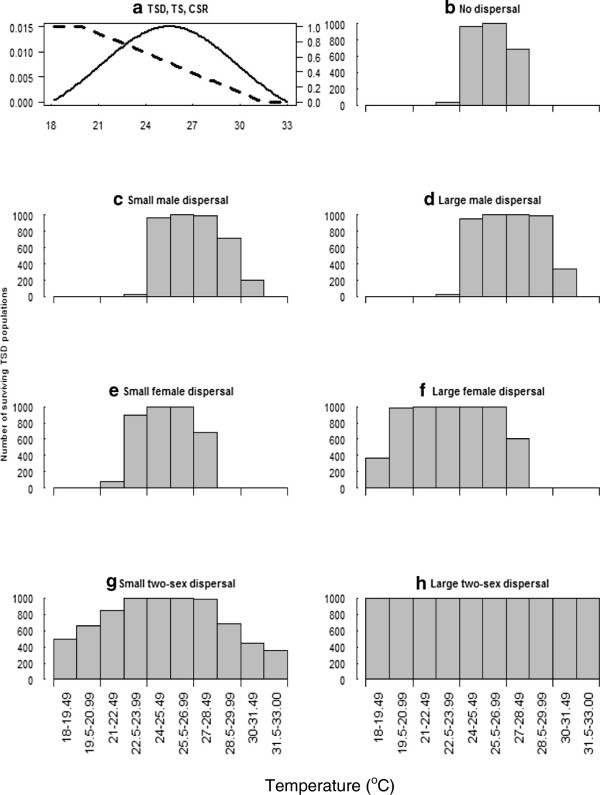
**Population persistence by temperature interval for temperature-dependent sex determination (TSD) populations. (a)** Temperature-dependent embryonic survival curve (TS) (black solid line) and cohort sex ratio (CSR) (black-dashed line) with intercept and slope parameters (α = 4.14, β = -0.147), for populations of reptiles with TSD. The unlabelled left axis represents juvenile survival and the unlabelled right axis represents the proportion of male hatchlings. **(b)** to **(h)** shows distributions of surviving populations of reptiles with TSD by temperature (°C) for dispersal conditions. The maximum population in each temperature interval is 1000.

We examined range limits under two relationships between cohort sex ratio (CSR) and air temperature. The first cohort sex ratio (CSR) curve was flat, with the offspring sex ratio at 0.5 for all air temperatures, describing the pattern for a GSD species (slope β = 0 and intercept of α = 0.5). CSR curve 2 represents a TSD species based upon the parameters derived for the painted turtle [20] with intercept α = 4.14 and slope β = -0.147 (see Figure 2a). The sex ratio produced at the long-term average air temperature for painted turtles is 0.6 (proportion of male offspring, [20]. Because we calculated population size for stable air temperatures, *p* did not fluctuate across years.

### Juvenile survival (*a*)

The number of juvenile males and females surviving the embryonic stage was sampled using a random binomial distribution based on the number of offspring produced of each sex and the juvenile survival rate. Juvenile survival rate was density dependent, *a*e^-*cN*^, where *N* is the total number of adult males and females in the population, *c* is the density-dependent constant (set to 0.001), and the baseline survival of juveniles (*a*) depended on temperature according to a normal distribution (the temperature-dependent embryonic survival (TS) curve, [Boyle M, Hone J, Schwanz L, Georges A: Under what conditions do climate-driven sex ratios enhance versus diminish population persistence? Ecology and Evolution. submitted].

The TS curve had a maximum baseline embryonic survival of *a*_*max*_ =0.015 (at 25°C) and a minimum baseline survival value of zero. The range of temperatures that produced non-zero baseline juvenile survival probabilities was 18 to 33°C. Survival rates are based on estimates from published values [44]. The temperature ranges for juvenile survival were similar to those reported for turtles with GSD [45] and TSD [15,46-48].

### Dispersal function

Several dispersal scenarios were evaluated owing to insufficient empirical information available to accurately parameterise dispersal in reptile species and variation among species in their dispersal tendencies. We explored three levels of dispersal: none, ‘small’ and ‘large’. However, these terms are relative as there is no readily available information to estimate what small or large dispersal would be in these species. For the ‘small’ amount of dispersal, juveniles had a higher probability of not dispersing and a lower probability of travelling large distances compared to juveniles with a ‘large’ amount of dispersal (Figure 1b). ‘Small’ and ‘large’ amounts of dispersal had three separate sex-based tendencies, i.e. male only, female only and two-sex dispersal. Under two-sex dispersal, males and females had equal probabilities of dispersing. Due to the stochastic natures of the juvenile sex ratio in each population and the dispersal function (below), the sex ratio of dispersers among cells was therefore also stochastic.

The dispersal function consisted of a probability density function (PDF) based on a fat-tailed (FT) dispersal kernel, which has been shown previously to best approximate dispersal in animals [49]. The FT dispersal kernel describes a scenario where most individuals disperse a short distance or do not disperse at all. In contrast, a small number of individuals disperse a long distance [49], as specified by the function:

(3) $$\mathrm{Pr}\left(\mathrm{dispersal}\;\mathrm{distance}\right)=1/\left(1+A*\left(D_{\mathrm{ij}}^{Z}\right)\right)\;$$

Pr(*dispersal distance*) is the probability that an individual moves a certain distance, D_ij,_ the distance moved between cells or populations. D_ij_ includes a distance of zero, thereby including the probability of not dispersing. Parameter A defines the distribution of dispersal differences, 1/A is the average dispersal distance, and Z is a shape parameter for the dispersal curve [50]. Pr(*dispersal distance*) represents the relative probability that an individual moves a certain distance between cells compared to other possible distances. This was converted to an actual or ‘real’ probability of dispersing each distance by dividing each relative probability by the sum of the values for all dispersal distances in the matrix (range of distances is 0–198 cells, see below).

Distances were calculated between cells (populations) as a von Neumann neighborhood, which counts the 4 cells immediately to each side of the focal cell as a distance of one, and the diagonal 4 cells as a distance of two [51]. Firstly, dispersal distance for each juvenile in each population was chosen based on the probabilities specified by the dispersal kernel. If a distance of zero was chosen the individual did not disperse and recruited to its natal population. If the distance was greater than zero, the target cell for dispersal was chosen randomly from all cells of the specified distance. Thus, dispersal from the cell in which the individual was born occurred in any direction on the matrix with equal probability. The edge cells of the matrix were defined as the ‘boundaries’ beyond which an individual could move no further. If a dispersal distance was chosen to which no cells corresponded (i.e. off the matrix), a new distance was chosen. Note that maximum possible dispersal distance varied among cells. Only the four corner cells of the matrix had a non-zero probability of dispersing a distance of the maximum 198 cells. Cells along the edge had higher maximum possible dispersal distances, but were constrained to disperse in fewer directions compared to cells in the center, which were more limited in distance but could disperse in more directions. We do not think this variation introduces much bias into the model as the parameter values we chose for the dispersal function specify exceptionally low probabilities of dispersing beyond 20–50 cells. An additional, small probability (0.1) of dispersal-related mortality in juveniles was incorporated into the dispersal algorithm. Dispersal related mortality did not increase with distance moved.

## Results

### GSD

**Figure 3:**
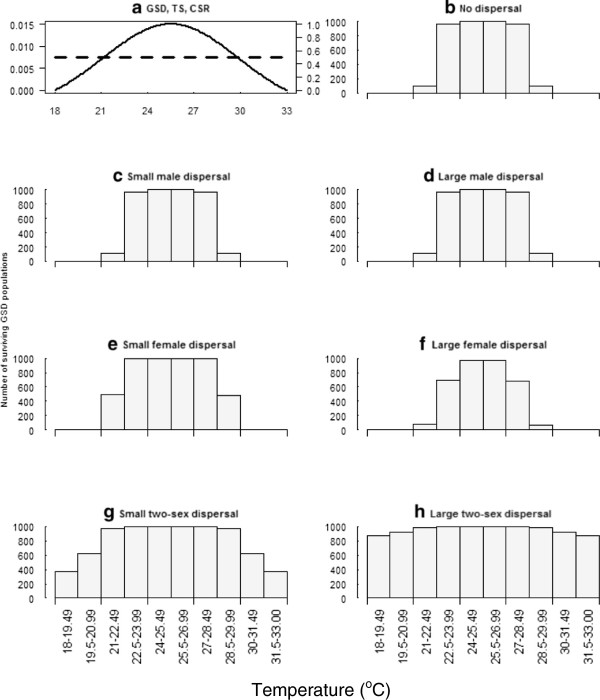
**Population persistence by temperature interval for genotypic sex determination (GSD) populations. (a)** Temperature-dependent embryonic survival curve (TS) (black solid line) and cohort sex ratio (CSR) (black dashed line) with intercept and slope parameters (α = 0.5, β = 0.0), for populations of reptiles with GSD. The unlabelled left axis represents juvenile survival and the unlabelled right y-axis represents the proportion of male hatchlings. **(b)** to **(h)** show the number of and distribution of surviving populations of reptiles with GSD by temperature (°C) for dispersal conditions. The maximum population in each temperature interval is 1000.

The limits of the range of GSD species (Figure 3) are not strongly influenced by the dispersal scenario, but seem instead to be primarily determined by the shape of the temperature-dependent embryonic survival curve (Figure 3a). Dispersal by males (Figures 3c, d) appears to have the same effect as no dispersal (Figure 3b) on population persistence.Dispersal does have modest effects on GSD populations when females disperse, but the effects differ between the small (Figure 3e) and large (Figure 3f) amounts of dispersal. The small amount of female dispersal bolsters populations at the edges of the range. This is presumably because of enhanced offspring production by immigrant females at the range margins that counteract the low juvenile survival there (Figure 3e). A large amount of female dispersal results in a reduction in the size of populations near the edge of the range. This is likely because females disperse frequently and far to populations where there are few to no surviving males, so the chances of reproducing are low. Increased dispersal of females out of near-edge populations reduces local reproduction. Thus, female dispersal can either enhance or diminish persistence near the edge of the range depending on how many females leave these populations and whether juvenile survival at the new population is high enough to provide a local supply of males.Dispersal by both sexes greatly expands the population persistence at most temperatures compared to other dispersal scenarios (Figure 3g). Indeed, for large two-sex dispersal (Figure 3h) the effects of dispersal are so large that most of the initial populations are maintained, except at the highest and lowest temperatures.

### TSD

When temperature influences the primary sex ratio, the results share some similarities with GSD, but also have several important differences. For both TSD and GSD species, there are large numbers of surviving populations around the central temperature intervals (from 24 to 28.5°C, Figures 3 and 2). In TSD species this 4.5°C temperature range represents a transition from a slightly male-biased sex ratio (around 60% male at 24°C) to a very female-biased sex ratio (around 80% female at 28.5°C). Without dispersal, TSD species have smaller ranges compared to GSD species. The range edge at warm temperatures is reduced modestly owing to decreased female fecundity where males are limiting. The range edge at cool temperatures is strongly reduced owing to low numbers of females, reducing overall offspring production.Under male dispersal, when males move into warmer areas they encounter increasingly larger numbers of females, and populations are no longer male limited. The importance of male limitation on female fecundity in influencing range limits is evident by comparing the results with and without male dispersal (Figure 2b, c, d). In the absence of dispersal (Figure 2b) warm populations are limited by the lack of males. In contrast, small and large amounts of male dispersal result in considerably greater population persistence in warmer areas (Figure 2c, d). Populations in the temperature intervals from 28.5 to 32.5°C (Figure 2c, d) benefit most from dispersal by males, as these very female-biased populations are able to persist in warmer areas where they do not in the absence of dispersal. Male dispersal cannot expand the range at the cold edge, where only males are produced, because dispersing males mostly encounter other males.Female dispersal produces the opposite result to male dispersal, expanding the range in the cool climates. This is because females dispersing to colder areas encounter a higher proportion of males than in populations of reptiles with GSD (Figure 3f). In contrast, if females disperse to warmer areas they encounter only other females, leading to consequent declines in reproduction and population persistence.Two sex dispersal (Figure 2g, h) increases ranges, similar to GSD species (Figure 3g, h). The shapes of the distributions of TSD and GSD species with two sex dispersal are very similar.

## Discussion

There is increasing evidence that factors such as population dynamics and dispersal strongly interact with abiotic factors such as climate in determining species ranges [3-8]. Species range limits can be strongly influenced by climatic effects on demographic parameters such as juvenile or adult survival, growth and reproduction [1,2]. Dispersal can play a key role in rescuing failing populations or creating “sinks” at range edges. Along range margins if the rate of colonisation via dispersal exceeds local extinctions, the range will expand. By contrast if local extinction events become more frequent than colonisations owing to extreme climates then the species range will shrink [8]. Here, we demonstrate the importance of the sex ratio in limiting species ranges, and explore its interaction with sex-specific dispersal.

Consistent with our previous work and Kallimanis [13], we found that, in the absence of dispersal, species with TSD are restricted in the climates in which they can persist compared to those with GSD owing to biased sex ratios in both warm and cool climates. In contrast to Kallimanis [14], we found that the restriction was greater in cool (male-producing) climates than in warm (female-producing) climates. The difference is owing to assumptions regarding male limitation [Boyle M, Hone J, Schwanz L, Georges A: Under what conditions do climate-driven sex ratios enhance versus diminish population persistence? Ecology and Evolution. submitted] – we assume that female fecundity is only reduced when males are less than 10% of the adult population.

In addition, we had several novel findings: 1) when both sexes disperse, the existence of biased sex ratios at extreme climates has almost no effect on limiting ranges; 2) male-only dispersal led to ranges covering areas where sex ratios were slightly male-biased to where they were heavily female-biased; 3) female-only dispersal led to ranges covering areas where sex ratios were slightly female-biased to where they were heavily male-biased; 4) dispersal scenario was more influential in driving range boundaries in TSD species compared to those with GSD. Our findings provide a clear distinction between populations of reptiles with GSD and TSD in the effect of dispersal at range boundaries, and by inference their responses to climate change. Understanding the sex-specific tendencies of dispersal will be imperative for predicting the possibility of range change for species with TSD.

Female-biased populations have been described as more likely to experience growth than populations with even sex ratios [28]. Furthermore, dispersal by male hatchlings is thought to have an important role in facilitating population persistence in increasingly feminised TSD populations under climatic warming [29]. We have demonstrated that male dispersal increases population persistence in female-biased populations located at warmer areas towards range margins (Figure 2c and d).

We show that population persistence at the colder (male-producing) edge of the range occurs only through female dispersal. Yet female dispersal has only been demonstrated in one GSD reptile, the alpine skink [30]. Skinks are considered to be inefficient at dispersal, and dispersal may occur over very short distances of a few metres [30]. In contrast, male marine turtles are considered to be very effective dispersers and may travel across oceans [36,37,39,52].

Two-sex dispersal has been poorly investigated across species [43]. Dispersal of both sexes in our model resulted in a potential for large ranges for both TSD and GSD species compared to no dispersal (Figure 3g, 3h, 2g, 2h). This expanded range led to populations persisting even in locations where juvenile survival was almost zero. In these scenarios, populations located at range edges are likely *sinks*, where the rate of production is below replacement level, and without sufficient immigration may become extinct [10]. Hence, under a climate change scenario, they may have a poor ability to produce their own migrants capable of expanding the range outwards.

Given the strong impact of the sex-bias and distance of dispersal on the results, these will be key behavioural traits to determine empirically when testing our theoretical predictions or making predictions about climate change. In addition, the distance of dispersal in a species must be considered with respect the spatial scale of its populations (e.g. distance is measured in number of populations that an individual can pass when dispersing). Our model makes the unique and testable prediction that, when comparing species of reptiles with pattern 1A of TSD, those species that have ranges where the sex ratios tend to be female biased will also to have male-mediated dispersal, while those that persist where the sex ratios tend to be male biased will have female-mediated dispersal.

While TSD species may persist in the short-term with biased sex ratios at range margins, an important caveat for our results is that populations at range margins (in extreme climates) may evolve in response to frequency-dependent selection on sex to produce more even sex ratios [53]. That is, local adaptation in the pivotal temperature (temperature at which a 50:50 sex ratio is produced) or maternal nesting behaviours could occur and reduce geographic variation in cohort sex ratios [54,55]. Existing geographical variation in nesting behaviour (for example, timing, nest depth and shade) may ameliorate the effects of local climate on nest temperatures, thus reduce spatial variation in survival and sex ratio [56]. However, recent work on the co-evolution of the pivotal temperature and dispersal suggests that local adaptation is often limited [11]. Owing to the demographic dominance of females, robust populations that produce a lot of dispersing offspring (i.e. populations with even or female-biased sex ratios) send their locally-adapted genes into other environments where the genes are maladapted and lead to biased sex ratios [11].

A critical assumption of our model is that each sex has an increasing benefit to population growth as the sex ratio becomes biased towards the opposite sex. However, as females become rarer this assumption may not hold anymore. For example, a low density of females has been demonstrated to result in male aggression and population collapse [57].

For our theoretical model to have explanatory power for the distributions of species with TSD, sex ratios must vary spatially. There is some evidence that sex ratios may be constant across the ranges of the painted turtle and the water dragon [54,55]. In contrast, sex ratios vary geographically in some sea turtles and crocodiles, although the variation is idiosyncratic and not linked to a continuous spatial component such as latitude [15,16]. A recent paper proposes that TSD reptiles do not exhibit balanced sex-ratios at the centre of their geographic distributions, and biased sex-ratios at range margins [16]. Future theoretical work should incorporate evolution of TSD traits as well as spatial variation in sex ratios that is more idiosyncratic across the range.

More broadly, testing our model and understanding the influence of climate of the ranges of species with TSD requires additional empirical data on the key demographic parameters across the ranges of TSD and GSD species. While data on dispersal are hard to collect, inferences about dispersal can be made from genetic analyses. Genetic analyses of sea turtles suggest that gene-flow is male-mediated and hence male-biased dispersal is the likely pattern [37-39,52]. Crude estimates of dispersal could be made by combining fixation index (F_ST_) (a measure of population differentiation owing to genetic structure) and demographic parameters. There is also some behavioural data which suggest that male-biased dispersal is the likely pattern in freshwater turtles [40-42], and this could be further investigated using techniques such as radio telemetry.

## Conclusions

Our theoretical model revealed that dispersal was more influential in determining the range limits of species with TSD than GSD. Male dispersal led to expanded species ranges across warm climates, where more females were produced, and female dispersal led to expanded ranges across cool climates, where more males were produced. Two-sex dispersal eliminated the influence of biased sex ratios on ranges.

## Competing interests

The authors declare that they have no competing interests.

## Authors’ contributions

MB contributed to the manuscript writing, modelling, and data analysis (including figures). LS contributed to manuscript writing and editing, modelling, and data analysis. JH contributed to the manuscript editing and developed the population equations underpinning the model. AG, contributed to the manuscript editing and provided the conceptual framework for the analysis. All authors read and approved the final manuscript.
